# Simple and rapid determination of tartrazine in fake saffron using the metal organic framework (Fe SA MOF@CNF) by HPLC/PDA

**DOI:** 10.1038/s41598-024-58825-x

**Published:** 2024-04-08

**Authors:** Ali salehi, Nabi Shariatifar, Gholamreza Jahed-Khaniki, Parisa Sadighara, Mohammad Hozoori

**Affiliations:** 1 Department of Environmental Health, Food Safety Division, School of Public Health, Tehran University of Medical Sciences, Tehran, Iran; 2 Saffron Institute University of Torbat Heydarieh, Torbat Heydarieh, Iran; 3 Drug Design and Development Research Center, The Institute of Pharmaceutical Sciences (TIPS), Tehran University of Medical Sciences, Tehran, Iran; 4 Department of Family and Community Medicine, Qom University of Medical Sciences, Qom, Iran

**Keywords:** Tratrazine, Saffron, MOF, CNF, Fe-single atom, D-μ-SPE, Environmental sciences, Health care

## Abstract

The present study of a novel metal–organic framework containing Fe single atoms doped on electrospun carbon nanofibers (Fe SA-MOF@CNF) based on dispersive micro solid phase extraction (D-μ-SPE) using HPLC–PDA for detection tartrazine in fake saffron samples was designed. The Fe SA-MOF@CNF sorbent was extensively characterized through various techniques including N_2_ adsorption–desorption isotherms, X-ray diffraction (XRD), scanning electron microscopy (SEM) and Fourier transform infrared (FTIR) spectroscopy. The specific area of surface of the sorbent was 577.384 m^2^/g. The study variables were optimized via the central composite design (CCD), which included a sorbent mass of 15 mg, a contact time of 6 min, a pH of 7.56, and a tartrazine concentration of 300 ng/ml. Under the optimum condition, the calibration curve of this method was linear in the range of 5–1000 ng/mL, with a correlation coefficient of 0.992. The LOD and LOQ values were ranged 0.38–0.74 and 1.34–2.42 ng/ml, respectively. This approach revealed significant improvements, including high extraction recovery (98.64), recovery rates (98.43–102.72%), and accuracy (RSDs < 0.75 to 3.6%). the enrichment factors were obtained in the range of 80.6–86.4 with preconcentration factor of 22.3. Consequently, the D-μ-SPE method based on synthesized Fe SA-MOF@CNF could be recommended as a sustainable sorbent for detecting tartrazine in saffron samples.

## Introduction

Nowadays, in all countries, food fraud has become a major issue and potential threat to consumers and food control organizations due to its direct consequences international trade and public health^1^. Therefore, it is very important to analyze food and find a fast and reliable solution to detect food fraud^2^. One of the most expensive foods in the world, which is known as the golden spice or red gold, is saffron which is obtained from the stigmas of *Crocus sativus L*. This spice is cultivated in a limited way in several countries due to special climatic conditions^3^. According to global statistics, more than ninety percent of the world's saffron is grown and manufactured in Iran^4^. Unfortunately, this spice has always been faced with various frauds by profiteers due to its high economic value. In recent years, one of the major frauds in saffron and its products is the use of artificial colors such as tartrazine, which has caused health concerns and economic losses for consumers^5^. Also, tartrazine well-known as FD & C Yellow No. 5, is a water-soluble azo color, which is allowed in some countries including the European Union (EU) and USA as an additive in a diversity of foodstuff like beverages, fruit juices, jellies, candies, gum, soup, ice cream and bakery products^6^. Although, azo colors are metabolized and removed through the liver and kidneys, many researches have presented these colors, especially tartrazine, can cause the numerous adverse effects such as neurobehavioral and hyperactivity disorders, certain types of tumors and cancers, asthma, urticaria and other allergic reactions by reducing the micro-flora and the forming the aromatic amines in the intestine^7^. Although Joint FAO/WHO Expert Committee on Food Additives (JECFA) evaluations in 2016 found that tartrazine in doses less than 10 mg/kg of body weight did not pose a health concern to people, multiple laboratory research have reported mutagenesis effects of tartrazine in rats and mice^8,9^. Despite all these issues, the presence of tartrazine at any quantity in *Crocus sativus* is considered fraud, and its detection is critical from an economic standpoint^5^.

In recent decades, many different methods have been investigated by researchers to detect adulteration in saffron. However, the most appropriate method is one that can effectively and expeditiously identify adulterants with minimal time, high precision, simple and cost-effective methodology. Considering the fact that there are many different types of food fraud, it is very challenging to usage a single technique to detect fraud in diverse food products, including saffron. Therefore, to obtain comprehensive information on the types of saffron adulterations, it is much more efficient to develop an integrated approach based on a combination of procedures^10,11^. Nowadays, the utilization of solid phase extraction techniques (SPE) in combination with nanocomposites as a novel methodology, have streamlined the processes of sample preparation, purification, preconcentration, and desorption. This approach has effectively obviated the need for membrane filters and solvent evaporation^12^. One of the SPE techniques that uses as sorbent in low quantities is D-μ-SPE. This approach is an advanced and specialized variation of conventional SPE in which the adsorbent disperses in the sample matrix and creates a strong interaction with the analyte without interfering with the sample matrix^13^.

MOFs (Metal–organic frameworks) are combinations of three-dimensional, which formed by connecting metallic clusters to organic linkers. These nanoporous compounds exhibit special characteristics like high porosity, structure of crystalline, large specific area of surface, high conductivity of electrical, and high stability of thermal, which give them a unique performance^14^. The presence of tunable nanoporous and a large area of surface has made MOFs provide more space for chemical interactions and absorption of molecules^15^. Based on previous literature, MOFs are widely applied in numerous applications, comprising gas absorption and storage, drug delivery, catalytic activity, sensing, food packaging, microbiology, and adsorption environmental and food pollutants^16–19^. In recent years, the use of MOFs as new adsorbents due to their unique characteristics, including high specific surface area, high adsorption capacity, high pore volume and porosity, and large accessible cavities, has been superior to conventional adsorbents such as silica, zeolites, and activated carbon^20,21^. In order to enhance the adsorption efficiency of MOFs, these three-dimensional structures can be combined with other nanostructures like carbon fibers.

CNFs (Carbon nanofibers) are one-dimensional carbon nanomaterials with diameters of 2–200 nm that have been widely used in various systems such as composite reinforcement^22^. Unlike other forms of nano-carbons, CNFs, with their outstanding electrical and thermal stability, and high surface area-to-volume ratio, are attractive substrates for the development of a wide range of materials, including enzymes, molecules, metals, and composites, notably MOFs^23^. MOFs and CNFs are suitable for analyte adsorption because of their intrinsic structural features, such as large porosity and surface area. Based on the Liu et al. study, the rate of electron transfer throughout the active sites is significantly enhanced by developing MOFs on CNFs^24^. Inspired by the aforementioned issues, it is anticipated that the development of MOF on CNFs will provide a stable nanostructure with high adsorption power. In the recent decade, researchers have developed a strong interest in using nanoparticles and nanocomposites as SPE sorbents for analyte extraction^25^. However, just a few studies have been undertaken on nano sorbents particulary metal organic framework doped on carbo nanofibers (MOF@CNF) combined with SPE methods for the detection of dyes in food samples.

In order to increase the speed of chemical reactions and absorption power, the use of catalysts is a key factor in the design of a nanocomposite^26^. As explained by Zhang et al., the catalyst based on single metal atoms (SACs) is one of the compatible catalysts with the physicochemical properties of nanomaterials^27^. Single atom catalysis technique is a process in which a metal single atom forms a chemical bond on composite surface to create single metal centers, which operate as active sites. In other words, each metal atom occupies a position on the surface of the catalyst, called a single active site. Therefore, with the existence of thousands of single atoms on the catalyst surface, there can be thousands of active sites. Furthermore, the formation of active sites depends on the high specific surface area of the desired composite^28,29^. Considering the favorable and high area of surface/volume ratio in MOF and CNF, implementing the SAC technique on MOF@CNF substrate is very effective. Regarding literature reviews, metal organic frameworks have demonstrated great performance in a wide range of SPE methods. Owing to the large specific area of surface and porosity of the MOFs, their incorporation as a solid adsorbent in SPE techniques can accelerate sample preparation and pretreatment^30^. Despite numerous researches on the usage of MOFs as adsorbents in the SPE method, few investigations have been conducted on MOFs coupled with the SPE techniques in the recognition of dyes in food sample^31^. Considering the need to detect tartrazine as a carcinogenic substance and the high economic value of saffron, it seems that using a fast, low-cost and high-precision approach can be useful and efficient. According to the studies and to our knowledge, this is the first research that a metal–organic framework containing single iron atoms has been dispersed on electrospun carbon nanofibers (Fe SA-MOF@CNF) which has been used as an adsorbent in the D-μ-SPE technique to detect tartazine in fake saffron samples. Therefore, the aim of the present study is to determine the presence of tartazine in saffron samples using the D-μ-SPE technique based on the new Fe SA-MOF@CNF nanocomposite and using HPLC–PDA (high-performance liquid chromatography equipped with a photodiode array detector).

## Materials and methods

### Reagents and materials

The pure saffron sample was provided from saffron institute of Torbat Heydarieh City, Iran. Tartrazine (Acid Yellow 23; FD&C Yellow No. 5) (99% purity) was obtained from medchemexpress (USA). N, N-dimethylformamide (DMF) (99.9%), ethanol (absolute ≥ 99.8%), methanol (HPLC grade; 99.8%), and distilled water (HPLC grade; 99.9%) were acquired from Merck Millipore (USA). Polyacrylonitrile (PAN, (C_3_H_3_N)n, Mw = 150,000) (98% purity), ferric nitrate nonahydrate (Fe(NO_3_)_3_⋅9H_2_O) (99%), zinc acetate dihydrate (Zn(CH_3_COO)_2_⋅2H2O) (98.8%), triethylamine (C_2_H_5_)_3_N (99%), terephthalic acid (C6H4-1,4-(CO2H)2) (98%), and triaminotriazine (C_3_H_6_N_6_) (99%) were obtained from Sigma-Aldrich Chemical Company (USA).

### Synthesis of Fe-single atom-MOF@CNFs

#### Preparation of carbon nano fibers (CNFs)

Carbon nanofiber, acting as a supporting substrate for the Fe-single atom-MOF was fabricated by electrospinning process with a slight modification according to KIM et al. and Tayebi-Moghaddam et al. methods^32,33^. To prepare the polymer solution, 10 g of polyacrylonitrile was dissolved in DMF (100 mL), and then the solution was homogenized using a magnetic stirrer at 300 rpm and 60 °C for 3 h. Next, the obtained polymer solution was injected into the electrospinning machine using a syringe pump (1 mL scale). The needle diameter and length were 0.25 mm and 4 cm, respectively. The injection flow rate was assumed to be 1 mL per hour. A positive voltage of 20 kV was applied. The distance between the drum collector and the needle was assumed to be 15 cm. Finally, the electrospun fibers were collected in aluminum foil and kept at − 20 °C till usage.

#### Preparation of MOF@CNF

The MOF@CNF was synthesized via the solvothermal technique modified by Tranchemontagne et al. method. At first, zinc acetate dihydrate (Zn(CH_3_COO)_2_ 2H_2_O) and terephthalic acid with a molar ratio of 2:1 were dissolved in 50 mL of DMF solution with 5 mL triethylamine under vigorous stirring at room temperature for 30 min. Then, the prepared CNF was added to mixture and it was again stirred for 1 h. The obtained solution was transferred to a teflon digestion bomb and then heated at 120 °C for 21 h by an autoclave machine in order to form bonds between MOF and CNFs. Subsequently, after cooling at room temperature, the solution was centrifuged for 10 min at 6000 rpm and then washed with methanol and DMF for twice times. Finally, in a vacuum oven, the product was dried at 60 °C for 12 h. The obtained powder was kept at − 20 °C for use in the next step^34^.

#### Preparation of Fe SA-MOF@CNF

After the synthesis of the MOF@CNF nanocomposite, according to previous studies with some modification, ferric nitrate nonahydrate (50% of the weight of the obtained powder) was added to MOF@CNF with 50 mL ethanol 70% and 2 mg triaminotriazine (as a nitrogen precursor) under vigorous stirring at 25 °C for 30 min. In next stage, the obtained solution was centrifuged at 6000 rpm for 10 min and via DMF was washed several times. After that, the achieved material was transferred to a vacuum dryer under the same conditions as the previous step. Finally, in order to pyrolysis and produce Fe single atoms, the dried powder by a laboratory furnace was heated for 1 h at 800 °C under protected nitrogen atmosphere^35^.

### Characterization of the Fe SA-MOF@CNF

The pore size distributions, pore volume and Brunauer–Emmett–Teller surface area, were analyzed by N_2_ adsorption–desorption isotherms using Micromeritics-ASAP 2020 adsorption analyzer. Fourier transform infrared (FTIR) spectroscopy (Bruker (USA)—Equinox 55) was performed for identify the functional groups and bonding arrangements in the wavenumber range of 4000–400 cm^−1^. Shape, morphology and particle sizes of the Fe SA-MOF@CNF were investigated by SEM device (MIRA3 TESCAN). In addition, the crystalline phases of sample was quantified by XRD analysis (Bruker d8-advance (Germany) diffractometer). Elements analysis of Fe SA-MOF@CNF was performed by Energy dispersive X-ray spectroscopy (EDX) technique. Zeta potential measurements were taken in deionized water with a zeta potential analyzer (DLS/Zeta Potential, Nano-flex). The thermal stability was investigated by a thermogravimetric analysis system (TGA; STA 6000, Perkin Elmer Corporation, USA) under a nitrogen atmosphere at a flow rate of 40 ml/min in a temperature range of 50–1000 °C with a heating rate of 10 °C/min.

### HPLC conditions

The stock solution of tartrazine at the level of 1 μg/L was provided in HPLC grade distilled water. Also, standard concentrations were carried out by diluting the stock solution in HPLC grade water. The chromatographic separation was performed by Waters Alliance e2695 HPLC system equipped with 2998-PDA detector at 425 nm using a Wonda Cract ODS-2 analytical column (5 μm, 4.6 mm × 250 mm) at 25 °C. The system mobile phase was consisted of three solvents including water (60%), acetonitrile (25%), and methanol (15%), with 1.0 mL/min rate of flow and 20 μL volume of injection^36^.

### Sample preparation and analytical procedure

The tartrazine separation from saffron samples were analyzed by the modified Alipanahpour et al. technique well-known as the D-μSPE-HPLC. Briefly, 1 mg of saffron sample was mixed with 100 mL of ultrapure distilled water. Then, Fe SA-MOF@CNF composite as the tartrazine sorbent was added to the samples in varying amounts of 5 to 25 mg. Next, in order to adsorption of tartrazine by Fe SA-MOF@CNF composite, the sample was sonicated in varying times (2–10 min) using a Bransonic® Ultrasonic Baths. In the next step, the obtained solution was centrifuged at 6000 rpm for 10 min and the supernatant was filtered through a 0.22 μm membrane filter. Finally, by diluting the supernatant, a concentration of 1 mg/L was obtained. pH was adjusted in desired values 3–11 by the addition of hydrochloric acid (1 moL/L) and sodium hydroxide (1 moL/L). The extraction variables including pH, Fe SA-MOF@CNF mass, content time and tartrazine concentration were optimized by RSM (response surface methodology) according to the CCD (central composite design)^37^.

### Adsorption selectivity

To determine the adsorption selectivity of Fe SA-MOF@CNF, 15 mg of Fe SA-MOF@CNF was exposed separately with 5 types of food additive dyes (sunset yellow, amaranth, quinoline yellow, brilliant blue, and tartrazine) under the same conditions (contact time: 5 min, pH = 7, and dye concentration: 5 µg/ml in each case). After reaching adsorption equilibrium, the concentrations of dyes in solution were determined by UV–Vis spectroscopy. Based on the Khoddami et al. (2020) study, the distribution and selectivity coefficients were calculated by the following formulas^38^:

$$\mathrm{Distribution \,coefficient}: D=\frac{Qe}{Ce}$$

$$\mathrm{Selectivity \,coefficient}: S=\frac{D1}{D2}$$

where Qe is the amount of nano sorbents and Ce is the equilibrium concentration; D1 represent the distribution coefficient of tartrazine and D2 is for sunset yellow, amaranth, quinoline yellow, brilliant blue, respectively; S is the selectivity coefficient of Fe SA-MOF@CNF.

### Elution conditions

According to the of Asfaram et al. (2018) study, in order to evaluate the effect of the eluting solvent in the D-μ-SPE procedure, 2–20 ml of methanol/hydrochloric acid (1 M) and methanol/hydrochloric acid (0.1 M) solvents in different concentrations (9:1, 7:3, 5:5, and 3:7) was examined^39^.

### Statistical analysis

By SPSS statistical software (IBM V. 21), all experiment data were evaluated, by using one-way ANOVA and the Duncan Multiple Range Test with a 95% confidence interval. To compare the study variables, the normality of the variables and homogeneity of variances were assessed using the Bartlett`s test and ANOVA. In addition, all study variables were optimized by the Response Surface methodology using Design Expert software (Stat-Ease Design Expert 13.0.5.0).

## Results and discussion

### Characterization of Fe SA-MOF@CNF

**Figure 1:**
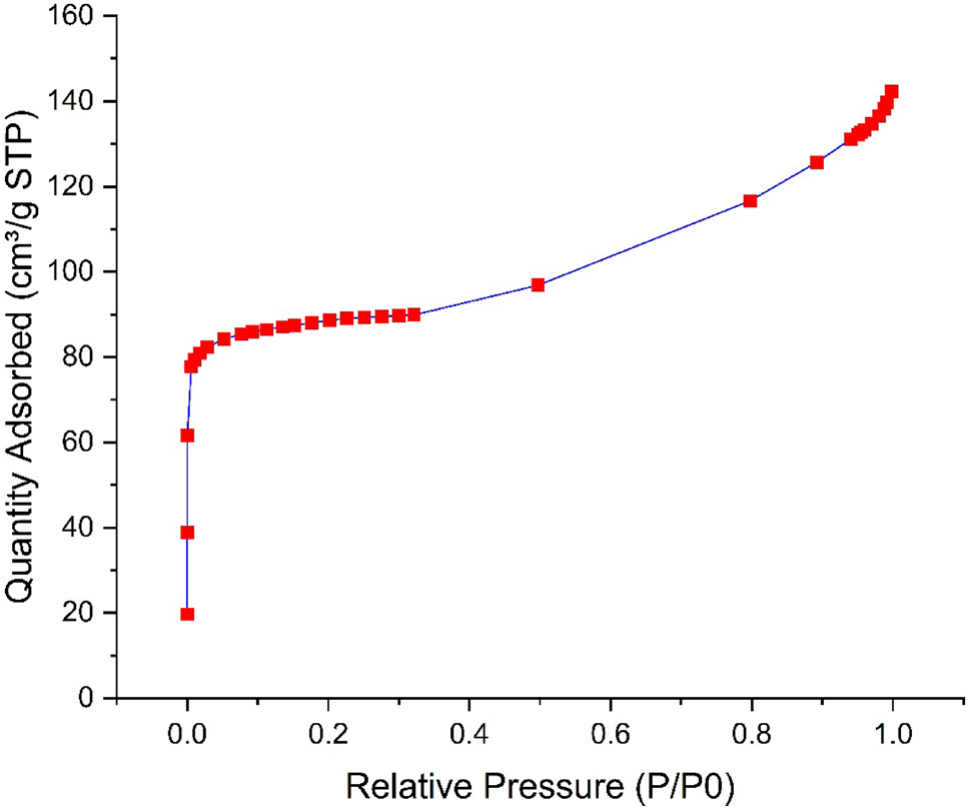
Diagram of N_2_ adsorption–desorption isotherms of Fe SA-MOF@CNF at constant temperature.

The Fe SA-MOF@CNF adsorbent was investigated using BET, FTIR, XRD, and SEM methods. The N_2_ adsorption–desorption isotherms of Fe SA-MOF@CNF at constant temperature according to Langmuir theory are presented in Fig. 1. The Langmuir scheme is based on multilayer adsorption of gas molecules on nanocomposite surfaces, which is mainly found in porous materials with pores smaller than 2 nm^40^. A summary of the BET reports are given in Table 1. The area of surface and pore volume of the Fe SA-MOF@CNF were 577.384 m^2^/g and 0.50489 cm^2^/g, respectively that was consistent with other researches such as Song et al.^41^. The large specific area of surface and acceptable pore volume indicate the high porosity of the desired nanocomposite, which leads to a higher amount of active surface sites and a larger capacity of adsorption in the Fe SA-MOF@CNF nanocomposite. The composite pores based on their size are classified to 3 categories including macropores (> 50 nm), mesopores (2–50 nm) and micropores (< 2 nm)^42,43^. The results of Halsey's standard model showed that the cumulative pore volume of desired nanocomposite was normally in the range of 1.7–to 30 nm. Metal catalysts tend to aggregate at the atomic level, but by anchoring them on suitable substrates with a large surface area, such as MOFs, a strong interaction among substrates and metal atoms is obtained^44^. The high surface area of a nanocomposite results in a greater number of active sites for chemical reactions and interactions. A high surface area can be attained by decreasing the particle size and rising the porosity^45^. Most investigations have found that nanocomposite surface areas of at least 200 m^2^/g produce satisfactory outcomes^46^. However, some studies have shown far larger nanocomposite surface areas. For example, in a study, Xie et al. were prepared a highly porous nanocomposite (Fe SAC-MOF-5) with a high external surface area (1651 m^2^/g) and an ultrahigh specific surface area (2751 m^2^/g)^35^.

**Table 1:** The Fe SA-MOF@CNF nanocomposite properties from Brunauer–Emmett–Teller (BET) analysis.

	CNF	MOF@CNF	Fe SA-MOF@CNF
Surface area (m^2^/g)			
Single point surface area	–	616.35	573.20
BET Surface Area	716	609.14	577.384 ± 6.61
t-Plot External Surface Area	–	–	146.587
t-Plot Micropore Area	–	–	230.5688
Langmuir Surface Area	–	–	911.2384
Pore volume (cm^2^/g)			
Total pore volume	0.512	0.576	0.50489
t-Plot micropore volume			0.21651
Pore size (nm)			
Adsorption average pore diameter	Pore size: 21.6	Pore size: 7.16	1.954
Desorption average pore diameter	–	–	1.926
Cumulative pore s (Halsey)	–	–	1.7–30
Median pore width	–	0.826	0.4231
Nanoparticle Size (nm)			
Average particle size	–	8.781	11.630

**Figure 2:**
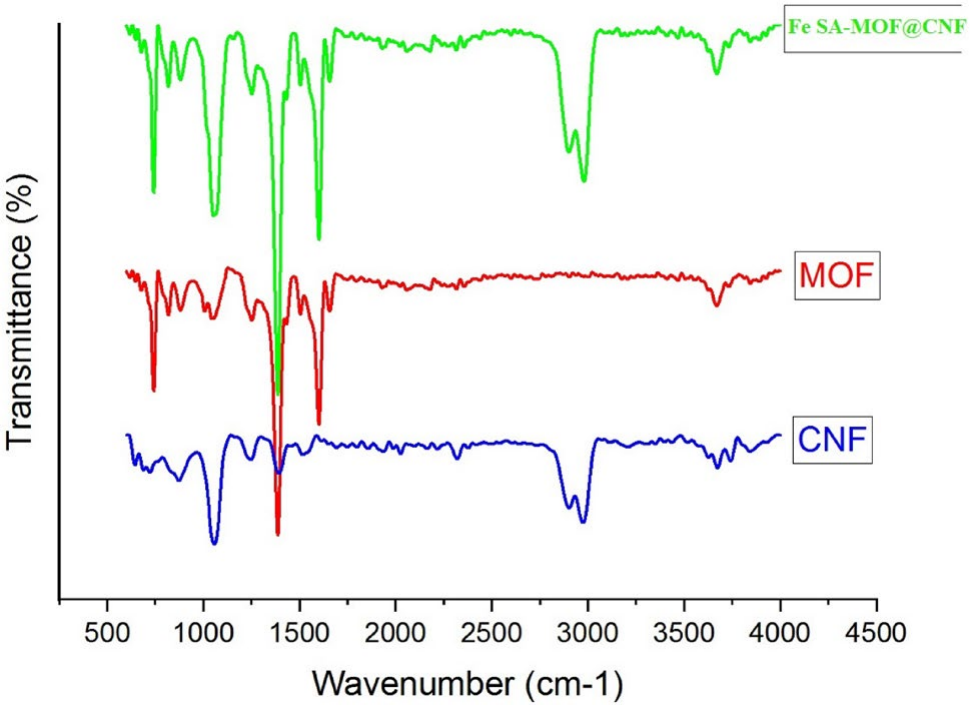
FTIR spectra of the CNF, MOF and Fe SA-MOF@CNF.

The FTIR spectra of the CNF, MOF and Fe SA-MOF@CNF are given in Fig. 2. Based on the IR spectrum of CNF, in the range of 715–880 cm^−1^, the strong and broad curvature peaks related to the C–C bonds were detected, verifying that the nanofiber structure was regularly formed. The C–O and O–H bands at 1060.71, 1249.69 and 1396.26 cm^−1^ attributed to stretching vibration of carbonyl and hydroxyl groups. The stretching vibration peaks of C-H are observed at 2906.32 cm^−1^ can be ascribed to alkyl group. Likewise, the peak at 2979.60 cm^−1^ can be attributed to carboxylic acid groups that confirming the successful polymerization of polyacrylonitrile. Furthermore, in the absorption region of 3675.81 cm^−1^, a weak C-N bond corresponding to the nitrile group is formed. The FTIR spectrum of MOF indicated a bending peak at 742.27 cm^−1^ attributed to the C-H group. A sharp peak appeared at 1390.4 cm^−1^, which was attributed to a C-H bond corresponding to aldehyde groups. The peak at 1603.37 cm^−1^ was ascribed to the amino group due to N–H stretching vibrations. In the IR spectroscopy of the Fe SA-MOF@CNF nanocomposite, five bands were observed. The first band is related to the bending C-H group in 744.41 cm^−1^. In the absorption region of 1068.42 cm^−1^, a relatively stretched C–O bond appeared, which was mainly attributed to the epoxy ring. Furthermore, a sharp peak was formed in the absorption of 1388.55 cm^−1^ belonging to the C-H bond due to the presence of aldehyde groups. The symmetric and asymmetric peaks in the region of 1506.19 and 1660.47 cm^−1^ were ascribed to C=O bonds in the hydrate and carboxylate groups, respectively. Another strong functional group on the surface of Fe SA-MOF@CNF is stretching N–O bond, which was formed at adsorbtion region of 1600.69 cm^−1^ in the nitrile group. The last peak in the range of 2904.39–2983.46 cm^−1^ is related to the stretching C-H band in the alkyl functional groups. It is noteworthy that three different peaks (744.41, 1388.55, and 1660.47 cm^−1^) with sharp adsorption band appeared by introducing a single Fe atom distributed over MOF rather than those of CNF alone. The FTIR spectra indicated that the Fe SA-MOF@CNF contains a greater range of functional groups than CNF^47,48^. We reasoned that anchoring a Fe single atom on carbon-derived MOF has the benefit of a variety of active sites formed over the surface catalyst, leading to enhanced capture of tartrazine molecules. In an innovative research work, Miri et al. designed magnetic Fe3O4@PDA@PANI core–shell nanoparticles as a new adsorbent for the simultaneous preconcentration of Sunset Yellow and Tartrazine using ultrasound-assisted dispersive micro-solid phase extraction. The FTIR results of the current research were comparable with the results of Miri et al.^49^.

**Figure 3:**
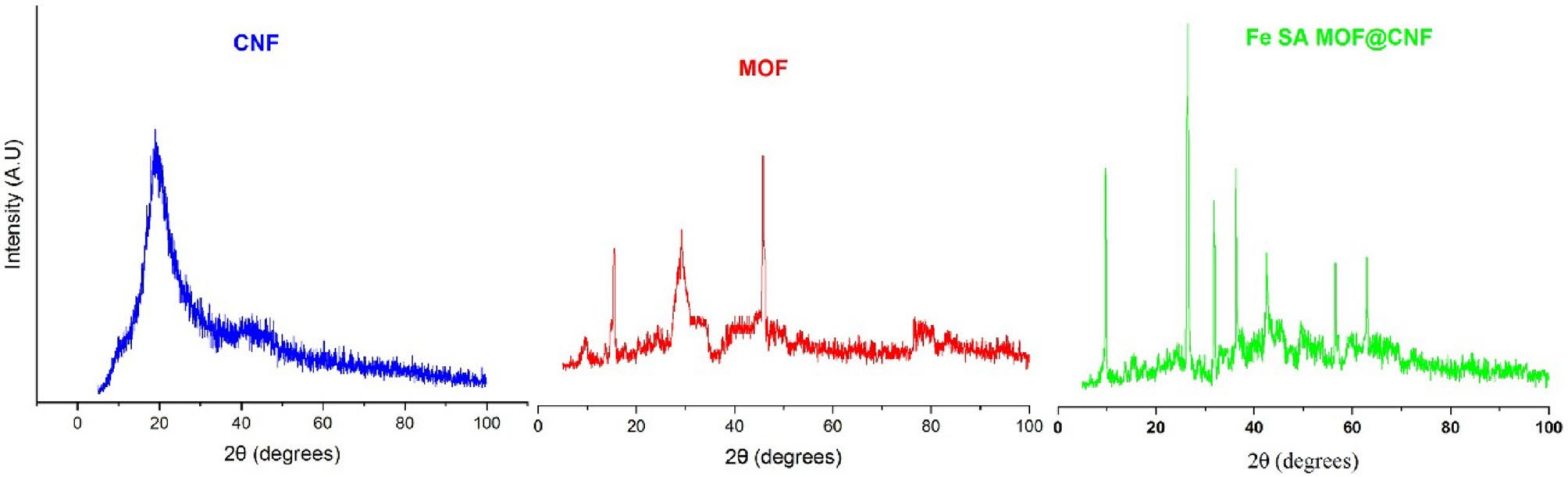
XRD patterns of MOF, CNF and Fe SA-MOF@CNF.

The XRD pattern of the MOF, CNF and Fe SA-MOF@CNF was shown in Fig. 3. The X-ray diffraction of CNF at 2θ = 18.37–20.43 showed a weak diffracted peak with low-degree crystallinity. The XRD pattern of MOF at angles 2θ = 15.63, 29.34, and 46.13 corresponds to (102), (118), and (179), respectively. The sharp peaks of MOF imply crystal structure development^50^. According to Fig. 2, the greatest crystal index was achieved by combining CNF with MOF and doping Fe single atoms during the pyrolysis process. All peaks of diffraction of the Fe SA-MOF@CNF were clearly characterized at 2 teta angles of 9.76, 26.32, 26.68, 31.84, 36.4, 42.56, 56.6, and 63.1, corresponding to (177), (259), (263), (152), (177), (111), (103), and (108), respectively. As inferred from Fig. 2 the sharp peaks clearly confirmed the nature of monophasic and polycrystalline of the Fe SA-MOF@CNF composite, which was consistent with Pradeep et al. and Pandey et al. studies^51,52^.

**Figure 4:**
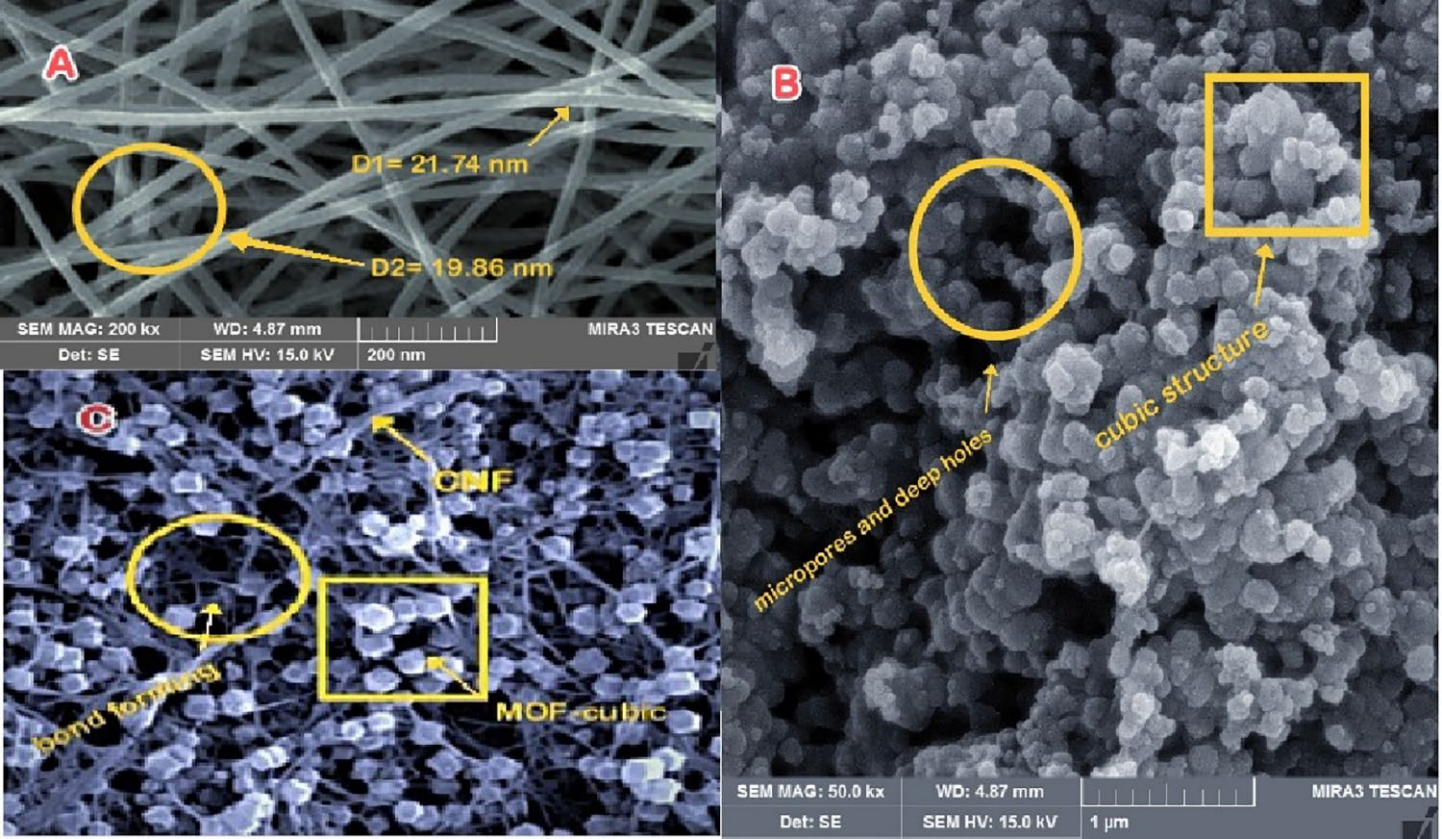
SEM images of (**A**) CNF, (**B**) MOF, and (**C**) Fe SA-MOF@CNF.

The microstructure and surface morphology of Fe SA-MOF@CNF were analyzed by SEM. SEM analysis (Fig. 4-A) illustrates the smooth and uniform surface of carbon nanofibers with a diameter of less than 30 nm. In the SEM image (Fig. 4-B), the cubic structure of the metal–organic framework composed of zinc and terephthalic acid can be clearly seen. Micropores and deep holes can also be found throughout the structure. This structure has the potential to accelerate electron transmission and molecule interaction^53^. Micropores are also required to produce active sites and molecule connections. Also, the presence of micropores is necessary to create active sites and connect molecules. The interaction of components in the carbon nanofiber substrate produced a heat-resistant and stable nanocomposite with a cubic structure^54,55^. Referring to the SEM analysis (Fig. 4-C), minimum Fe-related aggregates were formed, and the SEM analysis confirm the homogeneous distribution of elements forming bonds (N, C, and Fe). Referring to the SEM analysis (Fig. 4-C), minimal iron-related aggregates were formed, and the SEM analysis confirm the homogeneous distribution of the bond-forming elements. However, the final nanosorbent surface (Fig. 4-C) is rougher and more uneven than the CNF surface, owing to the inclusion of additional compounds such as iron metal. The results of SEM analysis in the study of Sohrabi et al. confirmed that following immobilization of Fe_3_O_4_-pyridine in MOF, the nanocomposite surface becomes rougher^56^.

As shown in Table 2, the SA-MOF@CNF sorbent is mainly composed of C, N, Fe, and Zn. The weight percentage of Fe was 6.21, indicating that some Fe particles may be distributed as single atoms on the nanosorbent surface.

**Table 2:** Energy dispersive X-ray spectroscopy (EDX) analysis of Fe SA-MOF@CNF.

Elt	Line	Int	Error	K	Kr	W%	A%	ZAF	Pk/Bg	Class	LConf	HConf
C	Ka	515.7	213.5632	0.8156	0.4293	73.73	83.13	0.5794	905.12	A	72.89	74.56
N	Ka	11.9	213.5632	0.0289	0.0199	15.60	14.32	0.0678	7.63	A	13.50	15.69
Fe	Ka	36.3	0.7851	0.0998	0.0457	6.21	1.65	0.8233	4.23	A	4.92	5.36
Zn	Ka	19.8	0.7851	0.0556	0.0357	4.46	0.90	0.7735	3.30	B	2.88	3.24
				1.0000	0.5306	100.00	100.00					

### Thermogravimetric analysis (TGA)

**Figure 5:**
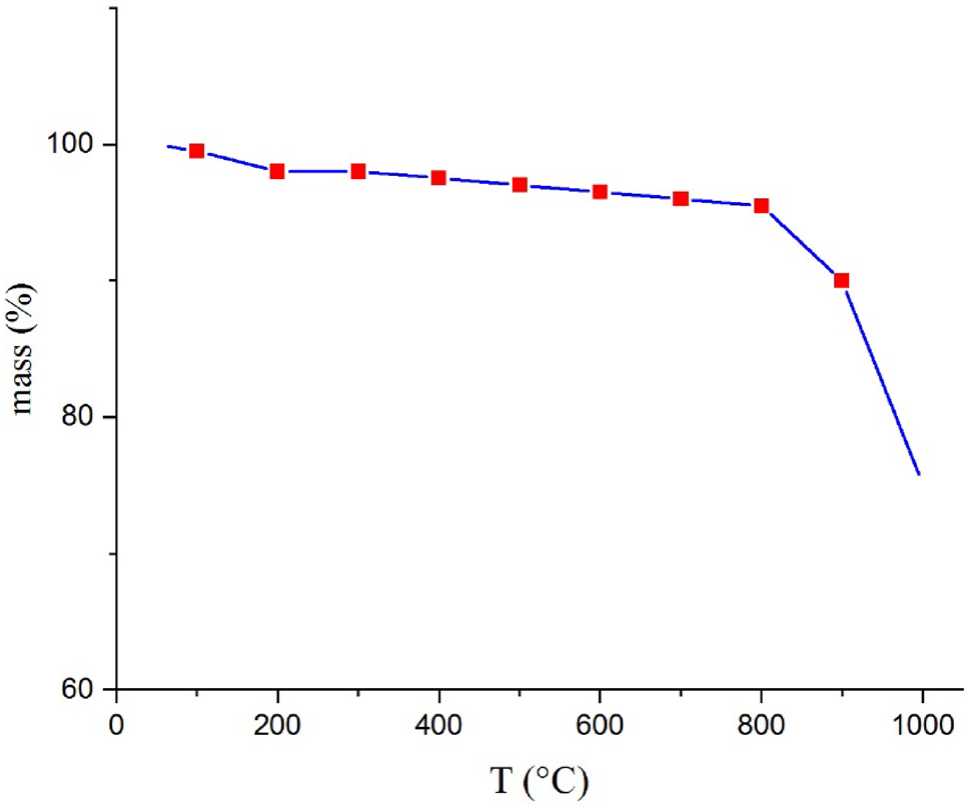
The TGA of Fe SA-MOF@CNF.

The thermal stability of SA-MOF@CNF is shown in Fig. 5. The TGA graph revealed a loss of mass sorbent in the temperature range of 100–200, which was attributed to dehydration caused by water evaporation^57^. In general, the thermogravimetric analysis demonstrated that the SA-MOF@CNF sorbent had substantial thermal stability up to 800 °C with a degradation rate of less than 5%.

### Experimental design and optimization of D-μSPE condition

To obtain the most optimal range of desired variables (pH, sorbent mass, contact time, and tartrazine concentration) to optimize tartrazine recovery based on the study of Ostovan et al., a total of 30 runs were randomly carried out using the RSM tool^58^. The design of the matrix of variables was defined by the CCD in 5 levels (− α, − 1, 0, + 1, and + α). Based on the equation α = 2^(K)/4^ and statistical methods, the alpha value was determined to be 2. Matrix design of the presented D-μ-SPE technique for tartrazine adsorption by Fe SA-MOF@CNF using HPLC–PDA based on central composite experimental design was given in Table 3. According to the results in Table 4, the effects of pH, tartrazine concentration, adsorbent dosage, and adsorbent contact time, as well as their interactions were determined together.

**Table 3:** Matrix design of the presented D-μ-SPEmethod for tartrazine adsorption by Fe SA-MOF@CNF using HPLC–PDA based on central composite experimental design.

Factor levels variables		− α (− 2)	Low (− 1)	Center (0)	High (+ 1)	+ α (+ 2)
A	pH (− log(H+))	3	5	7	9	11
B	sorbent mass (mg)	5	10	15	20	25
C	contact time (min)	2	4	6	8	10
D	Dye concentration (ng/ml)	100	200	300	400	500

	Factor 1	Factor 2	Factor 3	Factor 4	Response	
Run	A: pH	B: Tartrazine concentration	C: Sorbent mass	D: Contact time	Amount of tartrazine	
1	− 2.000	− 2.000	− 2.000	− 2.000	30	
2	− 2.000	− 1.000	− 2.000	− 2.000	60	
3	2.000	− 2.000	− 2.000	− 1.000	6	
4	− 1.000	− 2.000	− 2.000	− 2.000	31	
5	− 1.000	− 1.000	0.000	− 2.000	71	
6	1.000	− 2.000	2.000	− 1.000	19	
7	1.000	− 2.000	1.000	0.000	17	
8	1.000	0.000	0.000	− 2.000	38	
9	0.000	− 2.000	1.000	2.000	10	
10	− 2.000	0.000	− 1.000	− 2.000	95	
11	0.000	− 2.000	0.000	− 1.000	40	
12	0.000	0.000	0.000	− 2.000	40	
13	0.000	0.000	0.000	0.000	29	
14	0.000	1.000	1.000	− 1.000	48	
15	− 1.000	0.000	1.000	0.000	94	
16	0.000	1.000	− 2.000	0.000	30	
17	0.000	2.000	0.000	− 1.000	50	
18	2.000	− 1.000	− 1.000	0.000	11	
19	0.000	2.000	− 2.000	− 1.000	40	
20	− 1.000	0.000	1.000	1.000	88	
21	2.000	0.000	0.000	2.000	18	
22	1.000	0.000	0.000	− 2.000	39	
23	1.000	1.000	0.000	0.000	39	
24	− 1.000	1.000	1.000	2.000	106	
25	1.000	1.000	0.000	2.000	28	
26	− 2.000	0.000	1.000	− 2.000	105	
27	2.000	0.000	0.000	1.000	28	
28	2.000	2.000	1.000	0.000	46	
29	1.000	2.000	− 2.000	1.000	33	
30	1.000	2.000	− 2.000	− 1.000	42

**Table 4:** Homogeneity of variance and normality of study variables.

	pH	Sorbent mass	Contact time	Tartrazine concentration
Normality (Shapiro–Wilk)				
w	0.88	0.92	0.90	0.89
p-Value	0.17	0.44	0.22	0.25
Homogeneity of variances (Bartlett)				
Chi-squared	2.62			
p-Value	0.62			

**Figure 6:**
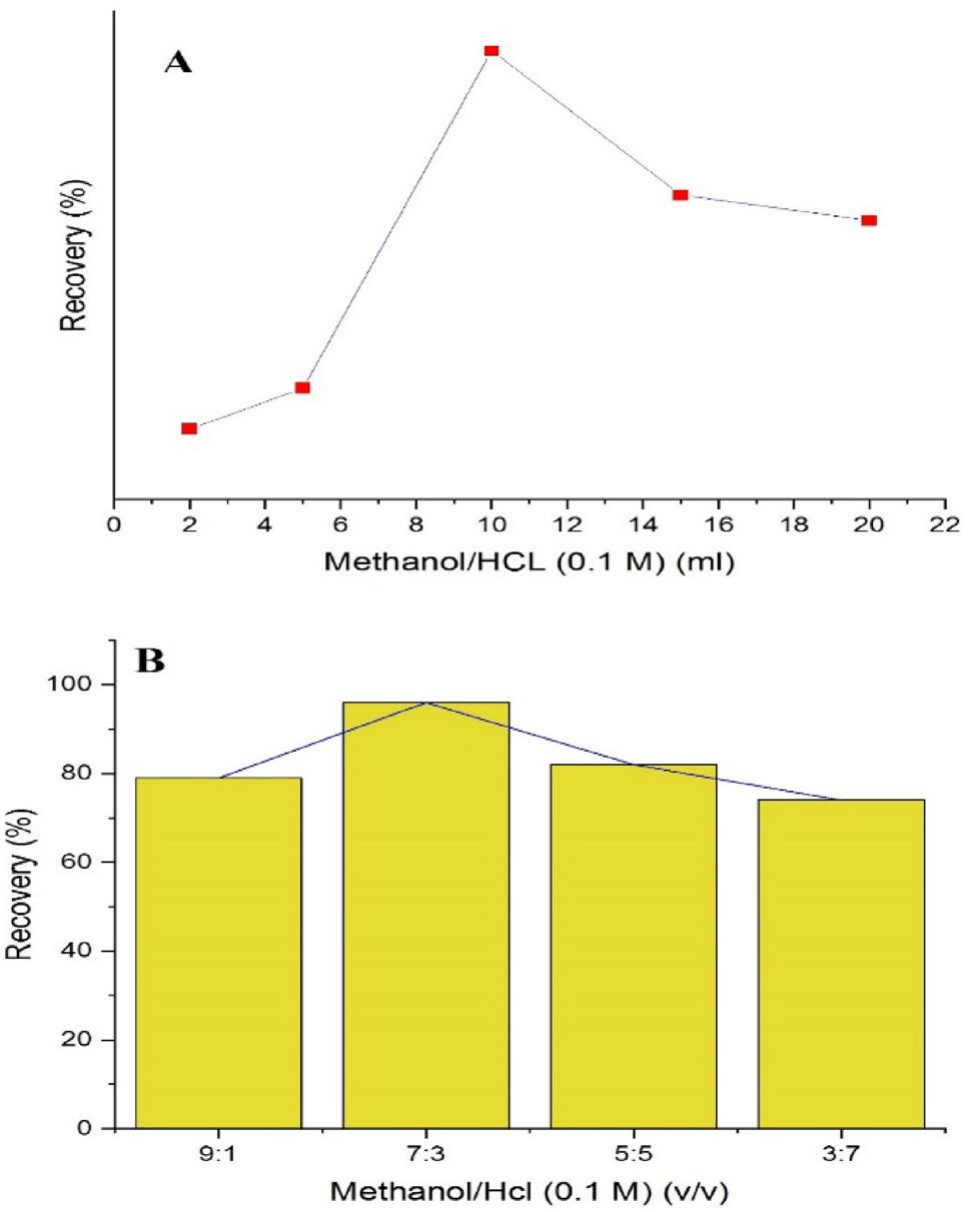
Effect of volume (**A**) of methanol/HCl 0.1 M solvent at different ratios (**B**) on the recovery of tartrazine in the D-μ-SPE method.

In the D-μ-SPE method, the type of eluting solvent is one of the most important factors influencing extraction efficiency and accuracy.As shown in Fig. 6, the eluting solution that eluted the most tartrazine was 10 mL of methanol/Hcl 0.1 M at ratio of 7:3 v/v.

In order to determine the sample solution's breakthrough volume, 1 mg of tartrazine was dissolved in 10, 50, 100, 150, and 200 ml of distilled water. Then the SPE procedure was followed^59^. The results revealed that the maximum extraction recovery for tartrazine was between 50 and 100 ml of sample volume. When the sample volume exceeded 100 ml, the extraction recovery decreased significantly. Thus, a 100-mL sample solution was chosen.

### Adsorption selectivity

Selectivity refers to the capability of nanosorbent to distinguish the desired analyte from other substances. In fact, selectivity is a measure of distinguishing the analyte signal from interfering signals^60^. As shown in Table 5, the adsorption selectivity of Fe SA-MOF@CNF toward tartrazine is significantly higher than those for other dyes. The high selectivity coefficient of Fe SA-MOF@CNF for tartrazine in the presence of sunset yellow, amaranth, quinoline yellow, and brilliant blue was most probably owing to increased physical and chemical bonding between Fe SA-MOF@CNF and tartrazine.

**Table 5:** Distribution ratio and selectivity coefficient of Fe SA-MOF@CNF toward tartrazine in presence of sunset yellow, amaranth, quinoline yellow, and brilliant blue under the same conditions (contact time: 5 min, pH = 7, mass sorbent:15 mg, and dye concentration: 5 µg/ml in each case).

S	D (dyes)^a^	D (tar)	
			Fe SA-MOF@CNF
16.42^b^	8.3	136.36^b^	Tar + SY
8.92	12.1	107.14	Tar + AM
9.48	7.53	71.42	Tar + QY
8.47	6.81	57.69	Tar + Bb

^a^Sunset yellow, amaranth, quinoline yellow and brilliant blue.

^b^Significance in the 95% CI.

### Effect of variables on tartrazine absorption

**Figure 7:**
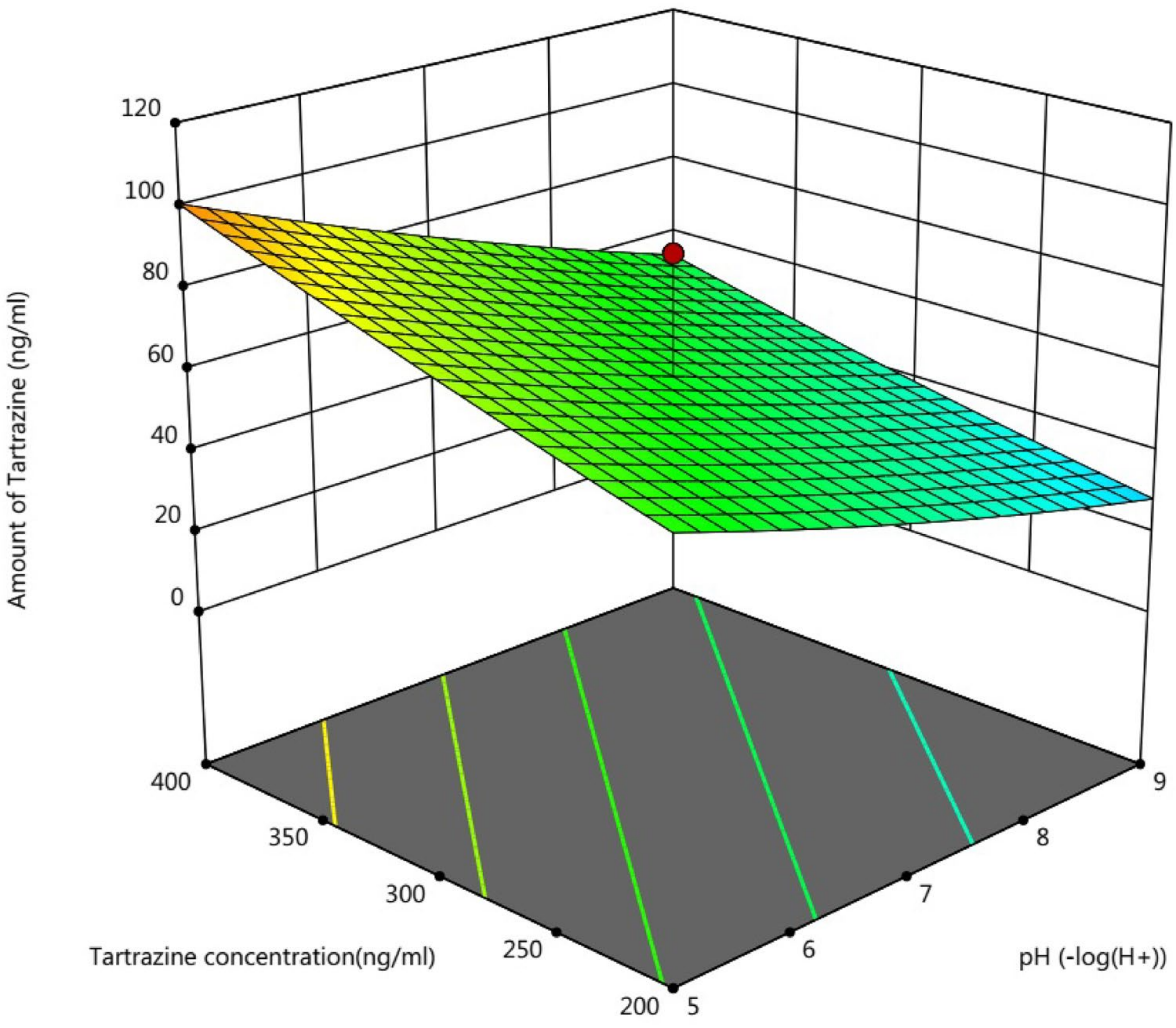
3D response surface plot of the effects of pH value and tartrazine concentration on the adsorption of tartrazine by the Fe SA-MOF@CNF adsorbent.

The research's findings revealed that alkaline pH had a substantial effect on tartrazine absorption (p < 0.001). The lowest quantity of tartrazine absorption by the Fe SA-MOF@CNF sorbent occurred at acidic pH. The amount of tartrazine absorption reached its maximum level when the pH was adjusted to 9. Meanwhile, there was no significant change (p > 0.05) in tartrazine absorption at pH greater than 9 (Fig. 7). The ionization of CNFs or the precipitation and elimination of terephthalic acid, and therefore the reduction of the interaction between vacant sites and tartazine molecules, are probably the causes of the reduction of tartrazine absorption at values of pH more than 9^61^. Moreover, the breakdown of active sites and the breaking of hydrogen bonds may be the cause of the decreased absorption of tartrazine at an acidic pH^62^.

The surface charge of Fe SA-MOF@CNF was determined by measuring its zeta potential at pH = 3–11. The zeta potential of Fe SA-MOF@CNF was positive at pH ≤ 6 and was negative at pH > 6. In optimum adsorption pH, zeta potential for Fe SA-MOF@CNF was − 17.64 mV. In this pH range, the tartrazine molecule most likely forms hydrogen bonds with the nano-adsorbent's hydroxyl functional groups^63^.

In the research conducted by Qin et al., for the result of tartrazine and ponceau 4R based on TiO_2_/electro-reduced graphene oxide nanocomposite, the optimal pH value of 7 was obtained^64^. However, in this study, the optimal pH was considered to be 7.56 by the central composite design technique.

The statistical analysis yielded findings indicating that the concentration of tartrazine positively impacted the tartrazine absorption by the Fe SA-MOF@CNF sorbent at a significant difference level of less than 0.01. As revealed in Fig. 6 the final amount of tartrazine raised along with the increase in tartrazine content in the synthetic samples. The outcomes revealed there was no significant difference (p > 0.05) in concentrations higher than 300 ng/L. Additionally, there was a positive and direct relationship between the quantity of tartrazine in the sample and the amount of adsorbent dosage, with a correlation coefficient (r = 0.9) at a significant difference level of less than 0.01. In other words, tartrazine absorption increased along with the amount of adsorbent. By increasing the sorbent volume, more active sites become available, allowing more tartrazine to be absorbed^65^.

**Figure 8:**
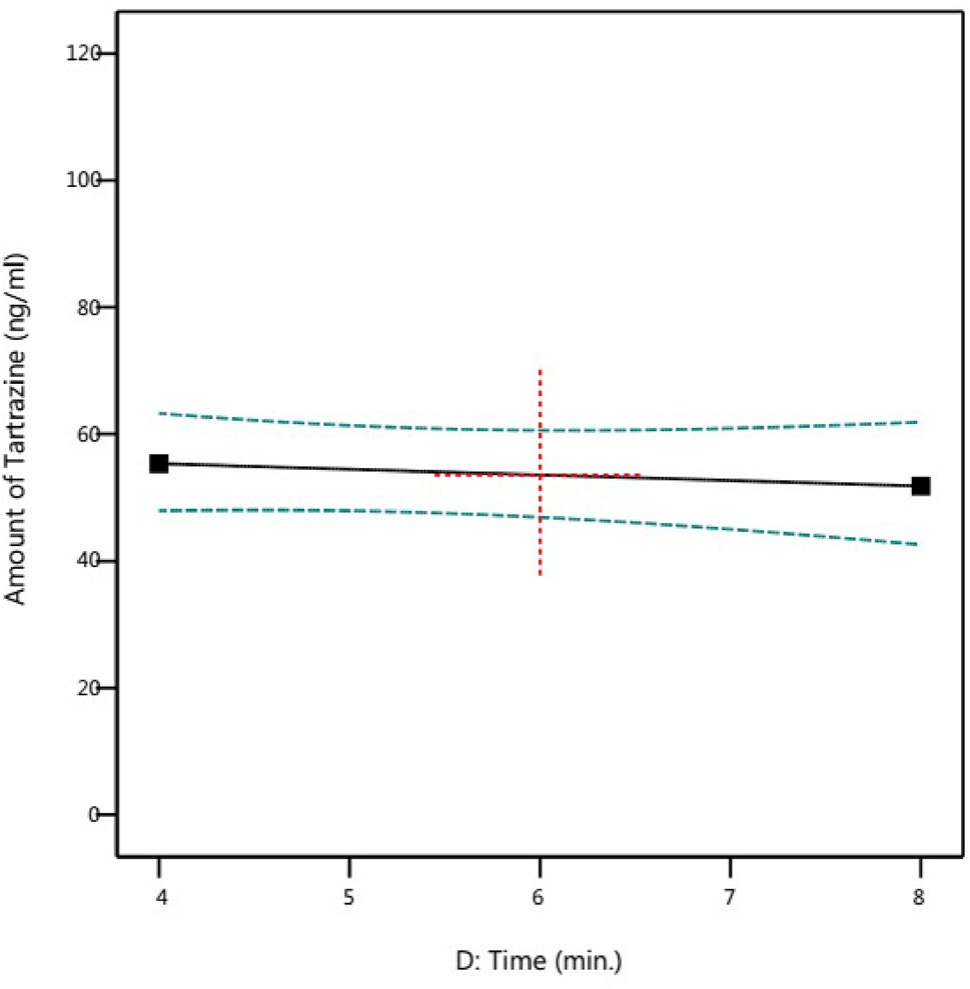
Effect of contact time on the adsorption of tartrazine by the Fe SA-MOF@CNF adsorbent (95% CI).

Figure 8 illustrates the correlation between the amount of extraction and the tartrazine contact time with the Fe SA-MOF@CNF sorbent. The findings showed that extending the time of tartrazine interaction with the sorbent had a negative effect on the amount of tartrazine extraction. In other words, by increasing the contact time of tartrazine with the desired adsorbent, more tartrazine was absorbed by the active sites on the adsorbent's surface, resulting in a decrease in the residual amount of tartrazine in the synthetic sample and its quantity in HPLC. The results demonstrated there was no significant difference (p > 0.05) in tartrazine absorption when the contact period was greater than 6 min. This suggests that the absorbent active sites in 6 min were saturated by tartrazine.

**Figure 9:**
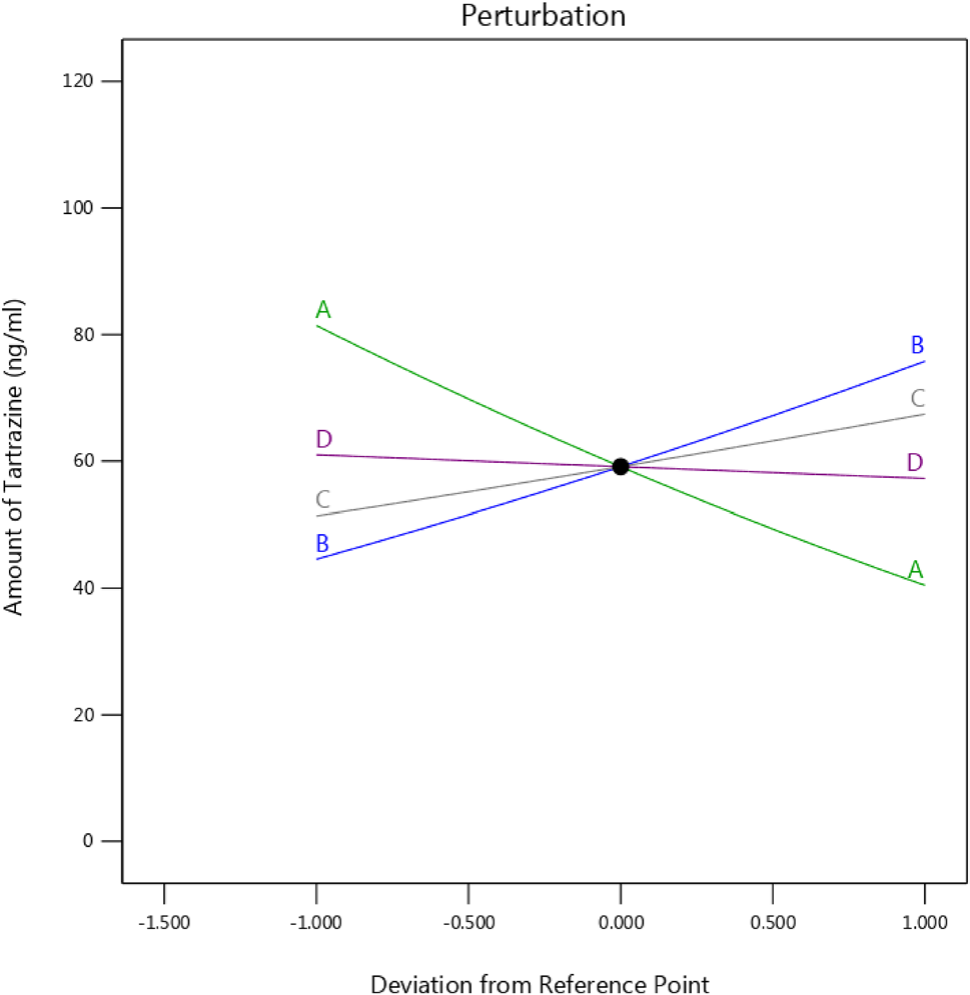
Perturbation plot of effects of study variables on tartrazine absorption by the Fe SA-MOF@CNF adsorbent at optimal conditions.

Based on the perturbation plot, the pH value is the most important factor affecting tartrazine absorption by the Fe SA-MOF@CNF sorbent (Fig. 9). The interaction among the hydrogen bonds and the functional groups in the composite containing carbon nanofibers and single active iron atoms is directly affected by the pH value^66^. Based on the RSM optimization model, the optimum values for the variables were as follows: a pH of 7.56, 15 mg of sorbent, 6 min of contact time, and a tartrazine concentration of 300 ng/mL.

### Model validation

Under optimal conditions, the D-μ-SPE approach based on Fe SA-MOF@CNF was validated for analysis of quantitative of spiked samples (Table 6). Tartrazine calibration curve in the range of 5–1000 ng/mL demonstrated the strong linearity with correlation coefficients value 0.992. The LOD (limit of detection) and LOQ values were 0.38–0.74 and 1.34–2.42 ng/mL, respectively. The enrichment item was designed as the ratio of the D-μ-SPE method curve slope to that of before extraction without pre-concentration, which ranged from 80.6 to 86.4 depending on the tartrazine concentration. It can be concluded that the good extraction efficiency and low limit of detection of Fe SA-MOF@CNF were owing to its homogeneous structure, the presence of numerous active adsorption sites, large specific surface, and high porosity.

**Table 6:** The parameters of analytical of D-μ-SPE -HPLC–PDA technique for tartrazine detection.

Validation parameters	Values
Linearity range (ng mL^−1^)	5–1000
Relative standard deviation (RSD %)	0.75–3.6
R^2^ (Correlation coefficients)	> 0.992
LOD (Limit of detection) (ng mL^−1^)	0.38–0.74
LOQ (Limit of quantification) (ng mL^−1^)	1.34–2.42
EF (Enrichment factor)	80.6–86.4
Extraction Recovery (ER %)	98.64
Pre-concentration factor	22.3

### The adsorption mechanism of tartrazine by Fe SA-MOF@CNF

According to the BET analysis, the Fe SA-MOF@CNF was classified as a mesoporous nanomaterial. Mesoporous nanomaterials have high absorption power due to their unique characteristics, such as a high specific surface area, a high porosity coefficient, and large pore sizes. In general, the current study suggests that the absorption mechanism of Fe SA-MOF@CNF was most probably physical–chemical. Chemical adsorption occurred through the formation of hydrogen bonds (C–H–N), whereas physical adsorption occurred through the trapping of tartrazine molecules in the Nano-sorbent's mesoporous^67^.

### Comparison of D-µ-SPE-HPLC–PDA method based on Fe SA-MOF@CNF with other techniques

A brief comparison between the present technique and other techniques for detecting and identifying the desired analyte in different matrices is presented in Table 7. The current investigation found that tartrazine extraction was accurate. In comparison to previous study, the D-μ-SPE technique based on Fe SA-MOF@CNF sorbent revealed a high relative recovery rate. In addition, the surface area of the current nanosorbent was greater than that reported in the literature. For example, in an interesting work by Oymak et al., a zirconium-based metal–organic framework (UiO-66(Zr)-(COOH)2) was synthesized as a sorbent for determining tartrazine in chewing gum, lemon-flavored icing glaze, and jelly samples; the surface area of the nanosorbent was just 79 m^2^/g^68^. The high surface area of the Fe SA-MOF@CNF sorbent is due to the small particles and favorable porosity of prepared MOF and presence of carbon nano-fibers, which is one of the advantages of this study^69^.

**Table 7:** Comparison of analytical data of the presented method with other reported methods.

Nano-materials*											
Surface area (m^2^/g)	Pore size (nm)	NP size (nm)	Preparation and analysis method	Sample	Optimum mass sorbent (mg)	LOD (ng ml^−1^)	LOQ (ng ml^−1^)	RSD%	R^2^	RR %	Refs.
CDs-MIP			Fluorescence spectroscopy	Saffron, tea bags, ice cream	–	0.7	–	3.2–4.5	0.996	97.4–104.1	^70^
–	–	< 10									
PTS-PPy NFsM			μ-SPE- HPLC–DAD	Wastewater	2	0.3–0.5	–	< 6.9	0.992	79.6–88.8	^71^
38.9	–	–									
CS-ZnO-NC			D-μ-SPE-HPLC–UV	Medical plants	12	0.06–0.089	0.201–0.297	1.02–5.69	> 0.993	96.6–105.4	^37^
Smooth	–	20–30									
Zr-BTeC			DSPE -spectrophotometer-UV–Vis	Gum, lemon glaze, jelly	10	11.6	–	3.8	–	93–110	^68^
79	1.2	–									
Cu-DTO MOF			Desorption and regeneration	Water	25	–	–	–	0.99	–	^72^
119.6	2–50	8.4									
Fe SA-MOF@CNF			D-μ-SPE-HPLC–PDA	Saffron	15	0.38–0.74	1.34–2.42	0.75–3.6	> 0.992	98.4–102.7	This work
577.384	1.95	11.6									

*CDs-MIP: carbon dots (CDs) embedded in molecularly imprinted polymer (MIP).

*PTS-PPy NFsM: P-Toluene sulfonate (PTS) doped polypyrrole (PPy) functionalized nanofibers mat (NFsM).

*CS-ZnO-NC: chitosan‑zinc oxide nanocomposite.

*Zr-BTeC: zirconium-based metal–organic framework.

*Cu-DTO MOF: Cu- coordinated dithiooxamide metal–organic framework.

### Real sample analysis

**Figure 10:**
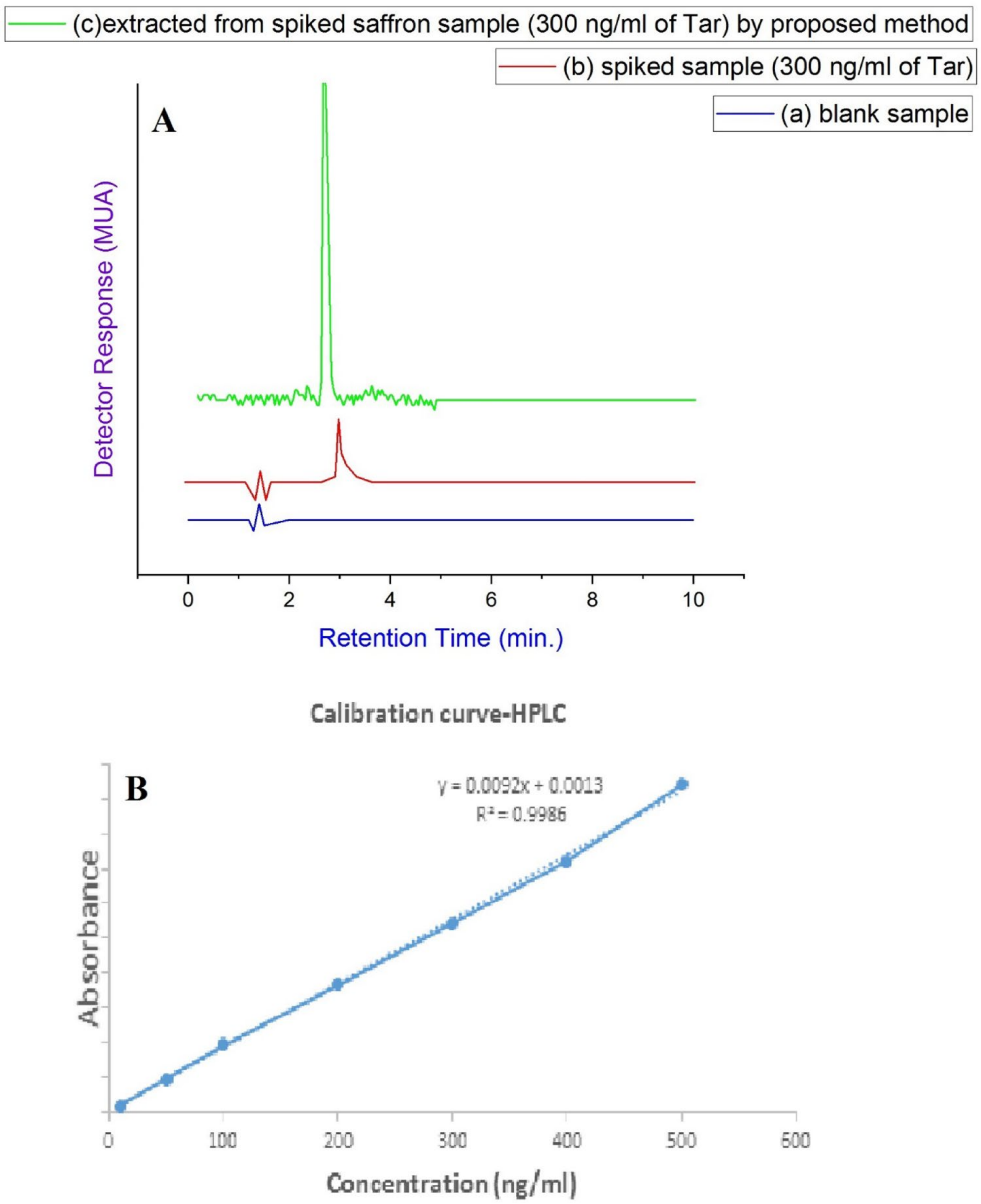
(**A**): Chromatograms of (a) the blank water sample, (b) spiked sample (300 ng/mL) without D-µ-SPE method, and (c) tartrazine extracted from real saffron sample spiked with 300 ng/mL of tartrazine by the D-µ-SPE-HPLC–PDA technique based on Fe SA-MOF@CNF at optimal extraction conditions (pH = 7.56, mass sorbent = 15 mg, contact time = 6 min, and tartrazine concentration = 300 ng/mL). (**B**): Calibration curve of tartrazine.

The tartrazine identity in a real saffron sample was determined by the HPLC–PDA coupled with the D-μ-SPE method, including Fe SA-MOF@CNF at levels of 0, 50, 100, 300, 500, and 1000 ng/mL. As revealed in Table 8, the recoveries varied from 98.43 to 102.72%, demonstrating that there is no significant matrix effect on the method's performance. The chromatograms of (a) blank sample, (b) the spiked sample, and (c) the extracted sample from Iranian saffron spiked with 300 ng/mL of tartrazine by the D-μ-SPE-HPLC–PDA procedure under optimal extraction conditions are revealed in Fig. 9. Tartrazine was identified as 418.83 ng/mL at a retention time of 2.81 min by the D-μ-SPE method. As shown in Fig. 10A-b, retention time of tartrazine detection in spiked sample (300 ng/mL) without Fe SA-MOF@CNF sorbent based on D-µ-SPE method was 3.01 min. The lower retention time in sample c indicated that the D-µ-SPE method based on Fe SA-MOF@CNF sorbent has been able to overcome the complexities of the real sample. It can be seen in Fig. 9, which the D-μ-SPE-HPLC–PDA-related peak was more Gaussian and ideal compared to the spiked sample peak without the proposed method. The results obviously suggested that the D-μ-SPE technique based on Fe SA-MOF@CNF is absolutely suitable for the detecting tartrazine in saffron samples.

**Table 8:** Tartrazine quantification in saffron sample at different concentrations using the D-μ-SPE-HPLC–PDA technique based on Fe SA-MOF@CNF.

sample	Added tartrazine (ng/ml)	Founded tartrazine (ng/ml)	Recovery (%)
Saffron	0	23.52 ± 1.75	–
	50	63.81 ± 1.25	99.81
	100	112.60 ± 0.95	98.43
	300	418.83 ± 2.5	100.37
	500	697.42 ± 1.65	98.65
	1000	1345.23 ± 2.86	102.72

## Conclusion

A newly sustainable metal–organic framework containing single iron atoms embedded on electrospun carbon nanofibers (Fe SA-MOF@CNF) was successfully synthesized using a solvothermal method as a nanosorbent in the D-μSPE procedure for the recognition of tartrazine in faked saffron samples. Based on the BET analysis, the nano-sorbent had a high specific surface and porosity. The formation of hydrogen bonds and complex chemical relations between the inorganic substance and the functional groups of the organic polymer matrix were confirmed by the XRD and FTIR patterns. The SEM analysis presented a considerable number of active sites inside a cubic homogenous structure. The applied models demonstrated a strong response of the Fe SA-MOF@CNF sorbent to the D-μ-SPE-HPLC–PDA technique. Following the RSM-CCD analysis, the most key factors in D-μ-SPE-HPLC–PDA of tartrazine was the pH. The Fe SA-MOF@CNF had no significant matrix effect on the D-μ-SPE performance. In conclusion, this investigation shown that the D-μ-SPE method based on Fe SA-MOF@CNF sorbent is appropriate for detecting tartrazine in saffron samples. It is expected that in the next studies, the influence of this nano-sorbent on the finding of additional food additive in food samples will be investigated.

## Data Availability

All data generated or analysed during this study are included in this published article.
